# Supplementary material for: Ssu72 phosphatase is a conserved telomere replication terminator
Source: EMBO J. 2019 Feb 22;38(7):e100476. doi: 10.15252/embj.2018100476 (PMC6443209; doi:10.15252/embj.2018100476)
Supplement: Supplementary file 1 — Appendix [file EMBJ-38-e100476-s001.pdf]

Appendix

Table of contents

1. Appendix Supplementary Methods
2. Appendix References
3. Appendix Figures S1-S7
4. Appendix Table S1

1. Appendix Supplementary Methods

Mass spectrometry

IP beads were incubated and boiled (95ºC, 5 min) in NuPAGE LDS sample buffer 1x (Invitrogen) containing 5 mM of DTT. Samples were allowed to reach room temperature and then cysteine alkylation was performed through the addition of acrylamide:bis-acrylamide (29:1) solution (Bio-Rad) to the final concentration of 7% (v/v). Samples were incubated for 5 minutes at room temperature and then were loaded on a 4-12% Bis-Tris NuPAGE gel (Invitrogen). Gel was stained using BlueSafe coomassie stain (NZYTech) and a gel image was acquired on ImageScanner III (GE Healthcare).

A slice of gel ranging between 75 and 63 kDa was excised and sliced into smaller pieces (1 mm x 1 mm). Destain, digestion and desalting was performed as previously described in (Luís *et al*, 2016), with minor changes: Gel pieces' dehydration steps were always performed in a vacuum concentrator (Labconco, Kansas City, MO USA); reduction and alkylation steps were performed before gel electrophoresis; and peptides were recovered from the C18 tips using 3 consecutive elutions, using increasing amounts of acetonitrile (50 %, 70%, and 90 % of acetonitrile in 0.1% Trifluoroacetic acid (TFA)). Eluted peptides were pooled together, dehydrated in a

vacuum concentrator, and resuspended in an aqueous solution of 0.1% formic acid (FA) (Solvent A).

Peptides were analyzed by nanoLC-MS using an Ekspert 425 nanoLC with cHiPLC (Eksigent, AB Sciex, Framingham, MA USA) coupled to a TripleTOF™ 6600 mass spectrometer (AB Sciex, Framingham, MA, USA). First, using 100% of Solvent A, peptides were loaded in a nano cHiPLC® trap column ChromXP™ C18 (200 µm internal diameter (i.d.) x 0.5 mm length, 3 µm particles, 120 Å pore size, Eksigent, AB Sciex, Framingham, MA USA) at 2 µL/min flow rate for 10 min. Then, using an analytical nano cHiPLC® column ChromXP™ C18 (75 µm i.d. x 15 cm length, 3 µm particles, 120 Å pore size, Eksigent, AB Sciex, Framingham, MA USA), the sample was resolved at 300 nL/min flow rate using a stepwise gradient of solvents A and B (0.1% FA in acetonitrile): 0-1 min, 95% A/5% B; 1-46min, 65% A/35% B; 46-48 min, 20% A/80% B; 48-54 min, 20% A/80% B; 54-57 min, 95% A/5% B; 57-74 min, 95% A/5% B. Peptides were ionized by a NanoSpray® III electrospray ionization source (AB Sciex, Framingham, MA USA) and the mass spectra were acquired in *information*-depended acquisition (IDA) mode: full spectra scan was acquired in the m/z interval from 400 to 2000 for 250 ms, and the 40 most intense ions were selected for subsequent MS/MS scans, which were acquired in the m/z interval from 150 to 1800 for 50 ms each. Only parent ions with a charge state between +2 and +5, and counts above the minimum threshold of 50 counts per second were selected for fragmentation by collision-induced dissociation (CID). Ions selected for MS/MS scan were excluded from further MS/MS analysis for 12 s. Fragmentation by CID was operated using rolling collision energy with a collision energy spread of 5.

Spectra were searched against Swiss-Prot database (downloaded in 10/2017, 5201 entries) containing all the reviewed protein sequences available for

Schizosaccharomyces pombe, three human keratin sequences (P04264, P35908, P13645), and the bovine trypsin sequence (P00761). The Paragon algorithm embedded in ProteinPilot 5.0 software (AB Sciex, Framingham, MA USA) was used to perform the database search using the following parameters: (1) sample type: identification, (2) cys alkylation: acrylamide, (3) digestion: trypsin, (4) instrument: TripleTOF 6600, (5) special factors: phosphorylation emphasis and gel-based ID, (6) species: none, (7) ID focus: biological modifications, (8) search effort: thorough, (9) detected protein threshold: >0,05. An independent false discovery rate (FDR) analysis was carried out using the target-decoy approach provided with ProteinPilot software and positive identifications were achieved using a global FDR threshold below 1%.

Gel overhang assay

Gel overhang assay protocol was adjusted with minimal changes from (Sfeir & de Lange, 2012). DNA (from fission yeast origin in figure Appendix S3 or human origin in figure Appendix S6) from different genotipes or treatments as indicated was either treated with *Mock* treatment or *Exonuclease I* treatment (100 Units) at 37 ºC overnight. DNA was then Phenol Clorophorm purified and Ethanol precipitated. Approximately 2 µg of digested *EcoRI* DNA in Fission yeast or *AluI* and *MboI* for human cells was run in either 1 % (fission yeast) or 0.6 % (human cells) agarose gels. Gels were dried with Bio-Rad gel dryer system (model 583). In-gel hybridization in native and denaturing conditions was labelled with a radiolabelled C-rich telomere probe and quantified for ssDNA at the telomeres.

2. Appendix References

Luís IM, Alexandre BM, Oliveira MM & Abreu IA (2016) Selection of an Appropriate Protein Extraction Method to Study the Phosphoproteome of Maize Photosynthetic Tissue. *PLoS One* **11:** e0164387 Available at: http://www.ncbi.nlm.nih.gov/pubmed/27727304 [Accessed May 14, 2018]

Sfeir A & de Lange T (2012) Removal of shelterin reveals the telomere end-protection problem. *Science (80-.).* **336:** 593–597 Available at: https://www.ncbi.nlm.nih.gov/pubmed/22556254 [Accessed March 8, 2013]

3. Appendix Figures

Appendix Fig S1

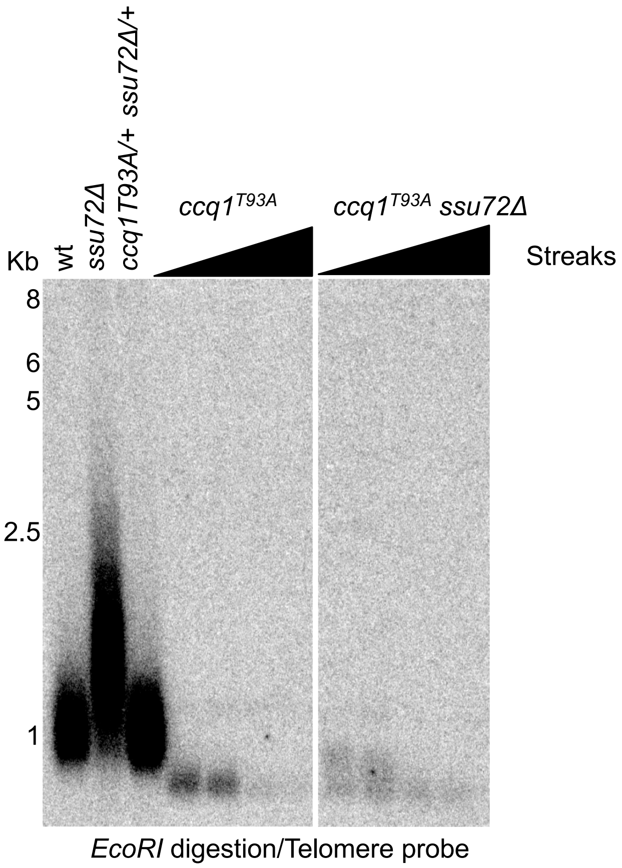

Appendix Fig S1: Phosphorylation of Ccq1 at Threonine 93 is required for telomere elongation in *ssu72* mutants

Diploid strains with the appropriate genotype were sporulated and streaked for different passages. Telomere length was measured in *EcoRI* digested genomic DNA by a telomeric probe.

Appendix Fig S2

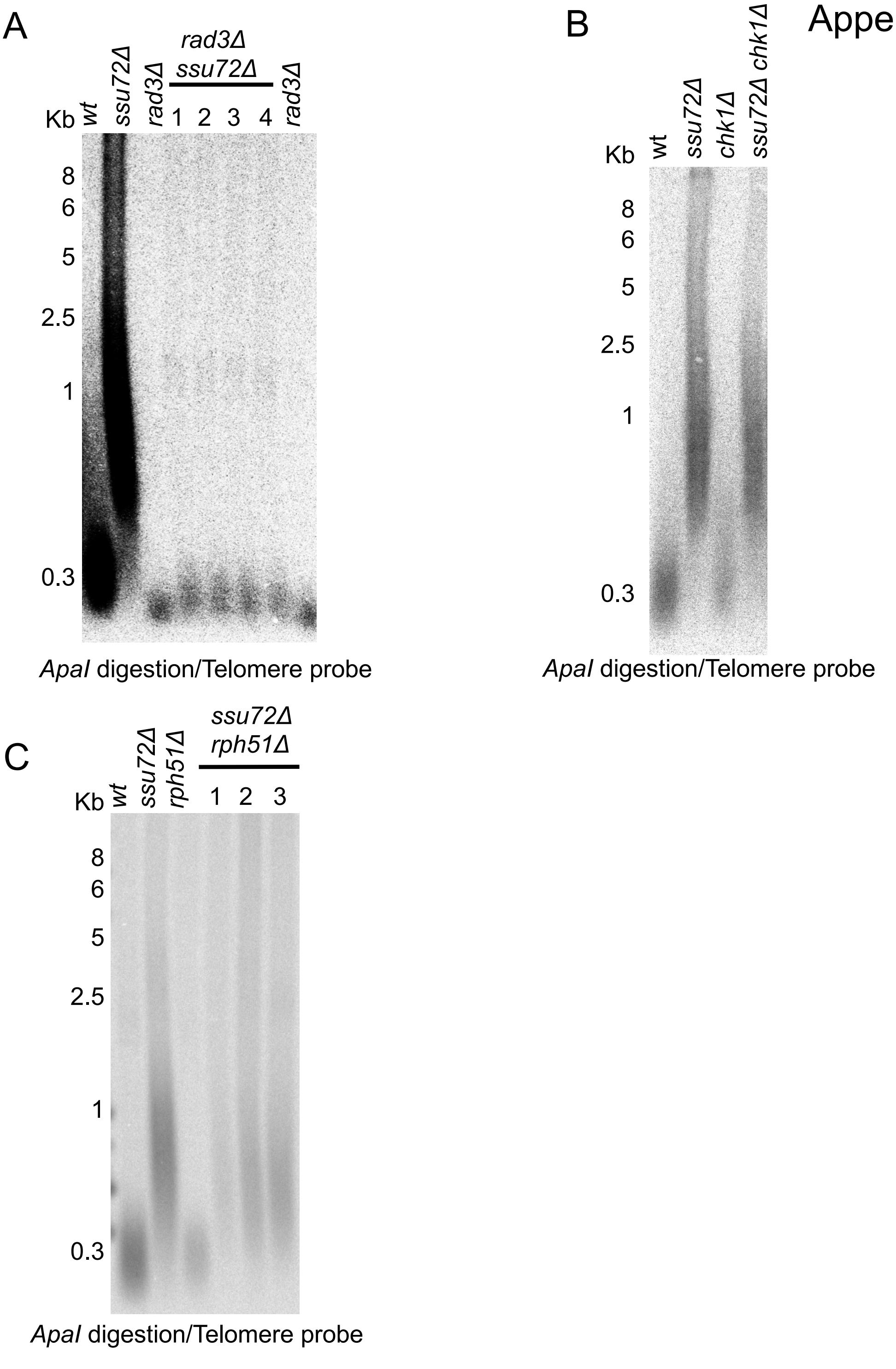

Appendix Fig S2: Telomere length in *ssu72*Δ mutants is *rad3*-dependent, but checkpoint and homologous recombination independent.

rad3Δ (A) chk1Δ (B), *rph51Δ* (C) single mutants or different colonies of *ssu72*Δ*-rad3Δ (A), ssu72*Δ*-chk1Δ (B), ssu72*Δ*-rph51Δ (C)* double mutants were constructed and telomere length was measured using Southern blots of *ApaI* digested genomic DNA using a telomeric probe.

Appendix Fig S3

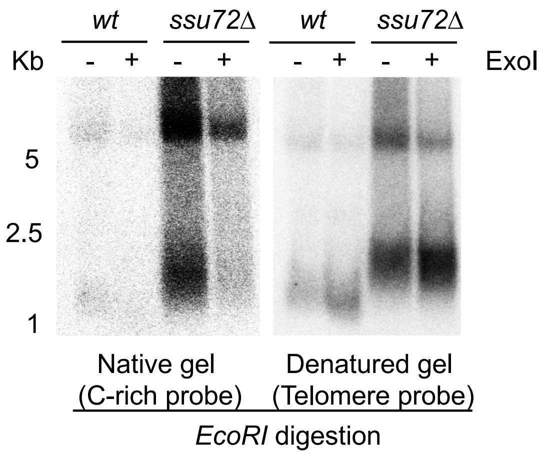

Appendix Fig S3: *ssu72Δ* telomeres exhibit longer terminal G-rich overhangs.
Genomic DNA was treated with exonuclease I as indicated and digested with EcoRI. In-gel hybridization under native and denaturing conditions was hybridized with a radiolabelled C-rich telomere probe

Appendix Fig S4

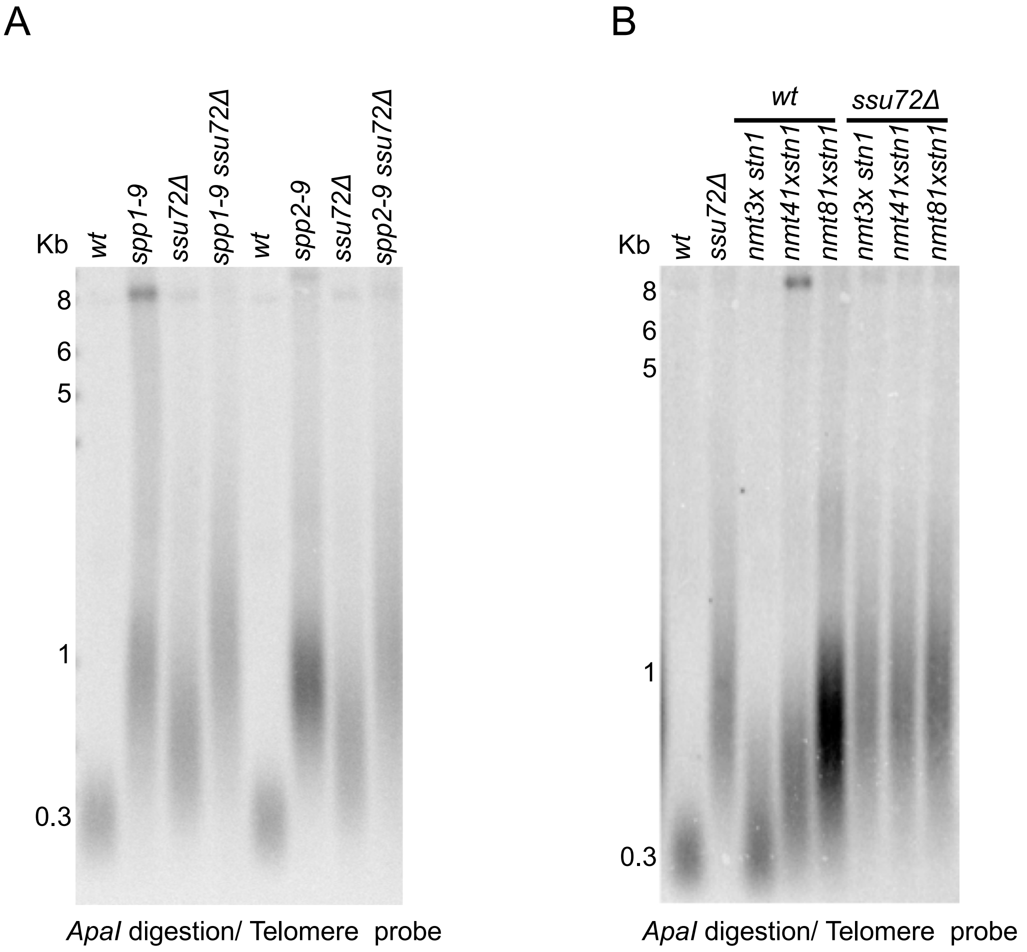

Appendix Fig S4. *ssu72*Δ telomere length is epistatic with polymerase alpha complex subunits.

A) Genomic DNA of single mutants of *pol1-13, spp1-9, spp2-9, ssu72*Δ and *wt or* double mutants *spp1-9 ssu72*Δ and *spp2-9 ssu72*Δ was isolated and telomere length was measured using Southern blotting of *ApaI* digested genomic DNA and telomeric probes. Temperature sensitive strains were grown at semi-permissive temperature by several generations and DNA was collected to carry out Southern Blot analysis.

B) Stn1 overexpression does not rescue telomere length defect of *ssu72*Δ*. stn1*$^{+}$ gene was expressed under3x (stronger), 41x and 81x (weaker) nmt1 promoter in *wt* or *ssu72*Δ background and telomere length was measured in *ApaI* digested genomic DNA.

Appendix Fig S5

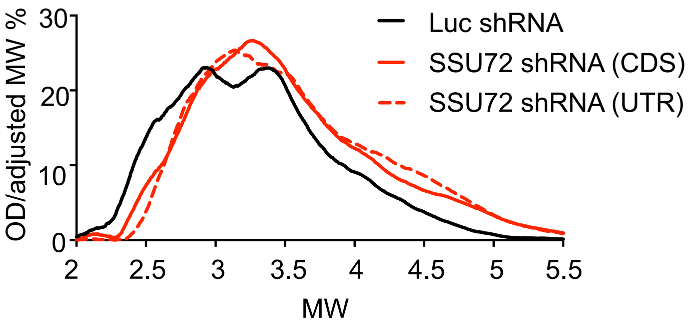

Appendix Fig S5: Densitometry of Telomere Restriction Fragment analysis of Figure 5A. Southern Blot densitometries from Luciferase shRNA, SSU72 shRNA (CDS and UTR) samples from figure 5A were acquired with imageJ and TRF analysis was carried out.

Appendix Fig S6

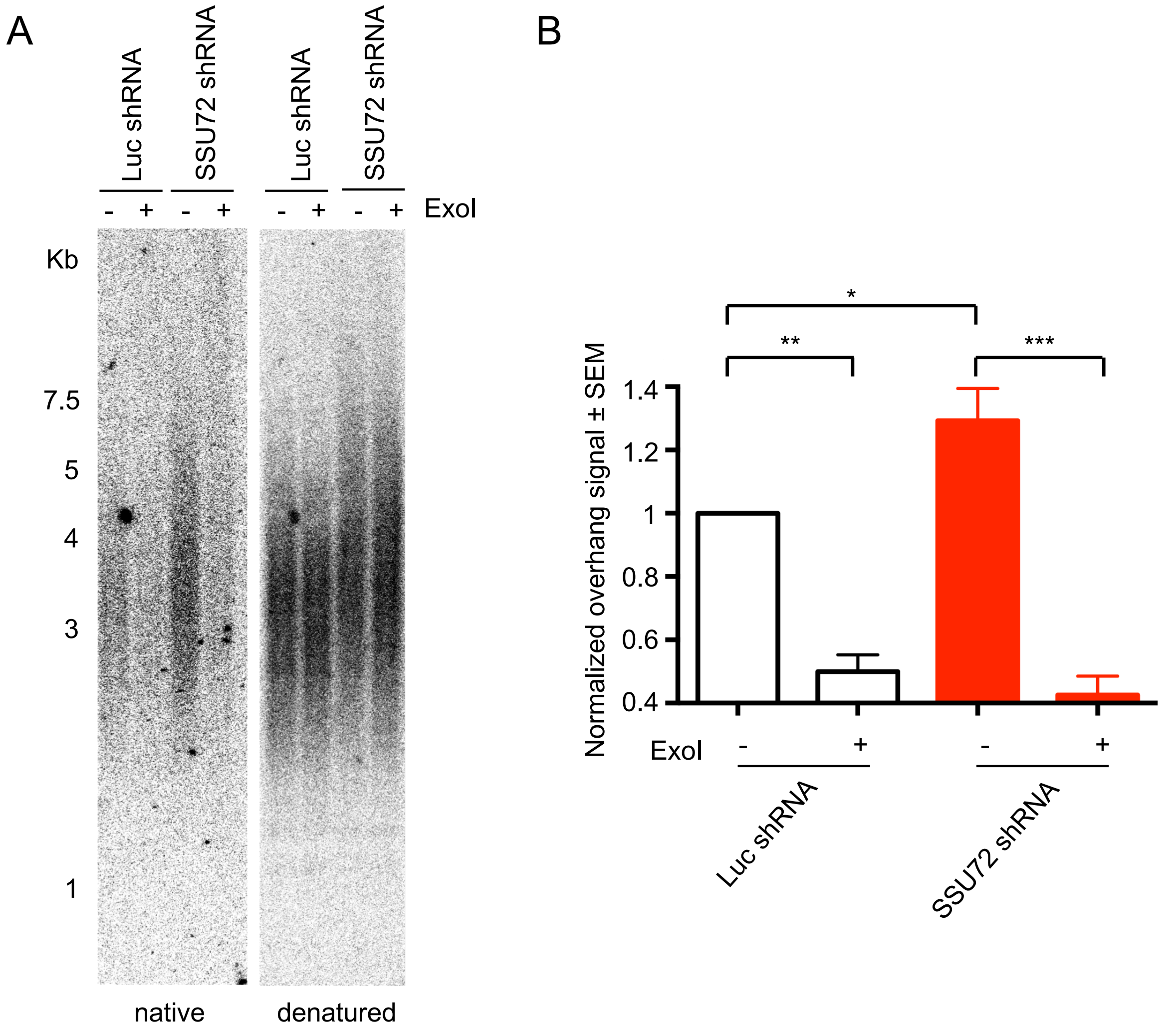

Appendix Fig S6: Telomere 3' overhang elongation upon SSU72 downregulation

A) DNA was treated with exonuclease I as indicated and digested with MboI and AluI. In-gel hybridization in native and denaturing conditions was hybridized using a radiolabelled C-rich telomere probe

B) Overhang intensity quantification from 4 independent experiments. Overhang signal was normalized with denatured gel and telomere length average. *p ≤0.05, **p ≤0.01 and ***p ≤0.001 based on a two-tailed Student's t-test to control sample. Error bars represent Standard error of the mean (SEM).

Appendix Fig S7

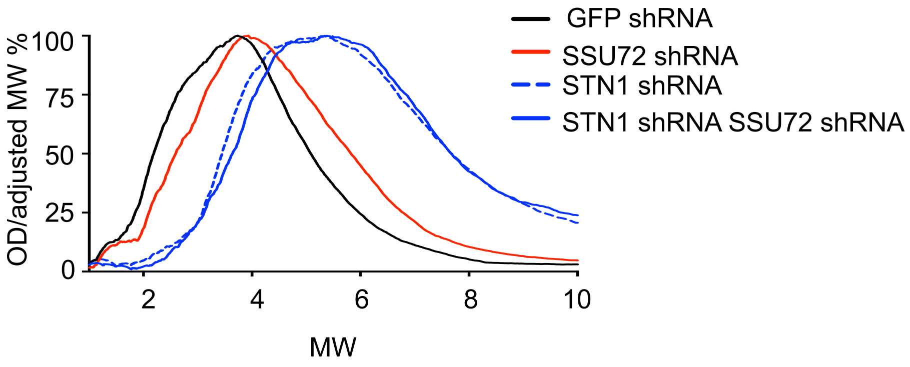

Appendix Fig S7: Densitometry of Telomere Restriction Fragment analysis of Figure 5B. Southern Blot densitometries derived from GFP shRNA, SSU72 shRNA STN1 shRNA for double SSU72 shRNA STN1 shRNA samples were acquired and TRF analysis was carried out

4. Appendix Table S1

strain number	Genotype		Origin
MGF 11	h+	ade6-M210 his3-D1 leu1-32 ura4-D18::KanMX6	This study
MGF 36	h-	ade6-M216 rif1::KanMX6	July Cooper's laboratory
MGF 106	h?	ura4-D18 cdc2-M68	Paul Nurse´s laboratory
MGF 168	h+	ade6-704 ura4-D18 leu1-32 rhp51::ura4+	Toni Carr´s laboratory
MGF 727	h+	ade6-M21? leu1-32 ura4-D18 his3-D1 rad3::NatMX6	This study
MGF 762	h+	ade6-M21? leu1-32 his3-D1 ura4-D18 cdc25-22	This study
MGF 2194	h-	ade6-M210 his3-D1 leu1-32 ura4-D18 ssu72::KanMX6	This study
MGF 2303	h?	ade6-M21? leu1-32 ura4-D18 his3-D1 rad3::NatMX6 ssu72::KanMX6	This study
MGF 2326	h-	ade6-M21? his3-D1 leu1-32 SPAC3G9.04::KanMX6 ura4-D18 rif1::HphMX6	This study
MGF 2377	h-	ade6-M210 his3-D1 leu1-32 ura4-D18 stn1::stn1 Nterminus tag nmt1-3X KanMX6	This study
MGF 2378	h-	ade6-M210 his3-D1 leu1-32 ura4-D18 stn1::stn1 Nterminus tag nmt1-41X KanMX6	This study
MGF 2379	h-	ade6-M210 his3-D1 leu1-32 ura4-D18 stn1::stn1 Nterminus tag nmt1-81X KanMX6	This study
MGF 2380	h+	ade6-M210 his3-D1 leu1-32 ura4-D18 stn1::stn1 Nterminus tag nmt1-3X KanMX6 ssu72:NatMX6	This study
MGF 2381	h+	ade6-M210 his3-D1 leu1-32 ura4-D18 stn1::stn1 Nterminus tag nmt1-41X KanMX6 ssu72:NatMX6	This study
MGF 2381	h+	ade6-M210 his3-D1 leu1-32 ura4-D18 stn1::stn1 Nterminus tag nmt1-41X KanMX6 ssu72:NatMX6	This study
MGF 2382	h+	ade6-M210 his3-D1 leu1-32 ura4-D18 stn1::stn1 Nterminus tag nmt1-81X KanMX6 ssu72:NatMX6	This study
MGF 2382	h+	ade6-M210 his3-D1 leu1-32 ura4-D18 stn1::stn1 Nterminus tag nmt1-81X KanMX6 ssu72:NatMX6	This study
MGF 2397	h-	ade6-M210 his3-D1 leu1-32 ura4-D18 ccq1:ccq1-FLAG KanMX6 rap1:HphMX6	This study
MGF 2398	h-	ade6-M210 his3-D1 leu1-32 ura4-D18 ccq1:ccq1-FLAG KanMX6 ssu72:NatMX6	This study
MGF 2399	h+	ade6-M210 his3-D1 leu1-32 ura4-D18 ccq1:ccq1-FLAG KanMX6	This study
MGF 2404	h-	ade6-M210 chk1::ura4 ade-6 leu1-32 ura4-D18 ssu72:NatMX6	This study
MGF 2435	h-	leu1-32 ura4-D18 his3-D1 trt1-G8-13myc:KanMX6	Toru Nakamura´s laboratory
MGF 2438	h-	leu1-32 ura4-D18 his3-D1 stn1-13myc:kanMX6	Toru Nakamura´s laboratory
MGF 2441	h?	ade6-M21? leu1-32 ura4-D18 his3-D1 trt1-G8-13myc:KanMX6 ssu72:NatMX6	This study
MGF 2443	h?	ade6-M21? leu1-32 ura4-D18 his3-D1 ten1-5FLAG-TEV-Avi-KanMX6 ssu72:NatMX6	This study
MGF 2547	h-	ade6-M210 his3-D1 leu1-32 ura4-D18 SSu72 C13S	This study
MGF 2548	h-	ade6-M210 his3-D1 leu1-32 ura4-D18 spp2.9::URA4	Teresa Wang´s laboratory
MGF 2549	h+	ade6-M210 his3-D1 leu1-32 ura4-D18 spp1.9	Teresa Wang´s laboratory
MGF 2550	h+	ade6-M210 his3-D1 leu1-32 ura4-D18 pol1:pol1-13	Teresa Wang´s laboratory
MGF 2552	h?	ade6-M21? his3-D1? leu1-32? ura4-D18 SPAC3G9.04::NatMX6 rad11::rad11-GFP (KanMX6r) taz1::taz1-mRFP (HphMX6)	This study
MGF 2555	h?	ade6-M210 his3-D1 leu1-32 ura4-D18 spp1.9 ssu72:NatMX6	This study
MGF 2560	h?	ade6-M210 his3-D1 leu1-32 ura4-D18 spp2::spp2.9 URA4 ssu72:NatMX6	This study
MGF 2564	h?	ade6-M210 his3-D1 leu1-32 ura4-D18 pol1:pol1-13 ssu72:NatMX6	This study
MGF 2579	h-	ade6-M210 his3-D1 leu1-32 ura4-D18 rep nmt41-empty vector Leu2	This study
MGF 2580	h-	ade6-M210 his3-D1 leu1-32 ura4-D18 rep nmt41-pol1oe vector Leu2	This study
MGF 2581	h-	ade6-M210 his3-D1 leu1-32 ura4-D18 SPAC3G9.04::KanMX6 rep nmt41-empty vector Leu2	This study
MGF 2582	h-	ade6-M210 his3-D1 leu1-32 ura4-D18 SPAC3G9.04::KanMX6 rep nmt41-pol1oe vector Leu2	This study
MGF 2587	h-	ade6-M210 his3-D1 leu1-32 ura4-D18 Ssu72 C13S Rif1::HphMX6	This study
MGF 2591	h-	ade6-M210 his3-D1 leu1-32 ura4-D18 ssu72::N terminus 13myc-ssu72	This study
MGF 2598	h-	rhp51::ura4+ leu1-32? ura4-D18? his3-D1? ssu72:NatMX6	This study
MGF 2605	h-	leu1-32 ura4-D18 ade6-M210 his3-D1 tpz1-K242R:hphMX6	Toru Nakamura´s laboratory
MGF 2669	h?	leu1-32 ura4-D18 ade6-M210 his3-D1 tpz1-K242R:hphMX6 ssu72:NatMX6	this study
MGF 2699	h-/h+	leu1-32/leu1-32 ura4-D18/ura4-D18 ade6-M210/ade6-M216 his3-D1/his3-D1 trt1::URA4 ssu72::NatMX6	This study
MGF 2700	h?	ade6-M21? his3-D1 leu1-32 ura4-D18 trt1::URA4 ssu72::NatMX6 streak 1	This study
MGF 2701	h?	ade6-M21? his3-D1 leu1-32 ura4-D18 trt1::URA4 ssu72::NatMX6 streak 2	This study
MGF 2702	h?	ade6-M21? his3-D1 leu1-32 ura4-D18 trt1::URA4 ssu72::NatMX6 streak 3	This study
MGF 2703	h?	ade6-M21? his3-D1 leu1-32 ura4-D18 trt1::URA4 ssu72::NatMX6 streak 4	This study
MGF 2707	h-	stn1-75	Alessandro Bianchi´s laboratory
MGF 2713	h?	ura4-D18 cdc2-M68 ssu72::NatMX6	Paul Nurse´s laboratory
MGF 2723	h+/h-	leu1-32/leu1-32 ura4-D18/ura4-D18 ade6-M210/ade6-M216 his3-D1/his3-D1 ccq1-T93A:NatMX6/ccq1+	This study
MGF 2733	h?	leu1-32 ura4-D18 ade6-M21? his3-D1 ccq1-T93A:NatMX	This study
MGF 2734	h?	leu1-32 ura4-D18 ade6-M21? his3-D1 ccq1-T93A:NatMX ccq1-T93A:NatMX ssu72::KanMX6	This study
MGF 2735	h-	ura4-D18 (leu1-32 ura4-D18 ade6-M210 his3-D1)? cdc25-22 ssu72-Myc tag Nterminus colony1	This study
MGF 2826	h+	ade6-M210? his3-D1 leu1-32 ura4-D18 pol1-Flag C-terminus (HphMX6) stn1-Myc (KanMX6)	This study
MGF 2829	h?	ade6-M210? his3-D1 leu1-32 ura4-D18 pol1-Flag C-terminus (HphMX6) stn1-Myc (KanMX6) ssu72::NatMX6	This study
MGF 2909	h-	ade6-M210? leu1-32 ura4-D18 his3-D1 stn1-13myc:KanMX6MX cdc25ts colony	This study
MGF 2912	h?	ade6-M210? leu1-32 ura4-D18 his3-D1 stn1-13myc:KanMX6MX cdc25ts ssu72::NatMX6	This study
MGF 3004	h-	ade6-M210 his3-D1 leu1-32 ura4-D18 Stn1s74D	This study
MGF 3005	h-	ade6-M210 his3-D1 leu1-32 ura4-D18 Stn1s74D ssu72:NatMX6	This study
MGF 3035	h?	stn1-75 ade6-M21? his3-D1? leu1-32? ura4-D18? ssu72::NatMX6	This study
MGF 3036	h?	stn1-75 ade6-M21? his3-D1? leu1-32? ura4-D18? ssu72::NatMX6	This study
MGF 3037	h-	ade6-M210 his3-D1 leu1-32 ura4-D18 Stn1s74D trt1::HphMX6	This study
MGF 3094	h+	cdc2as-M17-bsd leu1-32 ura-D18	Sato´s laboratory
MGF 3095	h?	cdc2as-M17-bsd leu1-32 ura-D18 ade6-M21? leu1-32 ura4-D18 his3-D1? stn1-13myc:kanMX6 ssu72:nat	This study
MGF 3096	h?	cdc2as-M17-bsd leu1-32 ura-D18 ade6-M21? leu1-32 ura4-D18 his3-D1? stn1-13myc:kanMX6 ssu72:nat	This study
MGF 3097	h?	cdc2as-M17-bsd leu1-32 ura-D18 ade6-M21? leu1-32 ura4-D18 his3-D1? stn1-13myc:kanMX6 ssu72:nat	This study
MGF 3097	h?	cdc2as-M17-bsd leu1-32 ura-D18 ade6-M21? leu1-32 ura4-D18 his3-D1? stn1-13myc:kanMX6 ssu72:nat	This study
	h+	ade6-M210 ura4-D18 leu1-32 SPAC30D11.10::KanMX6	Bionner´s library
	h+	ade6-M210 ura4-D18 leu1-32 9SPBC2F12.04::KanMX6	Bionner´s library
	h+	ade6-M210 ura4-D18 leu1-32 SPBC17D1.06::KanMX6	Bionner´s library
	h+	ade6-M210 ura4-D18 leu1-32 SPAC926.09c::KanMX6	Bionner´s library
	h+	ade6-M210 ura4-D18 leu1-32 SPAC3G9.04::KanMX6	Bionner´s library

Table S1. Strains used in this manuscript
