# Supplementary material for: Ssu72 phosphatase is a conserved telomere replication terminator
Source: EMBO J. 2019 Feb 22;38(7):e100476. doi: 10.15252/embj.2018100476 (PMC6443209; doi:10.15252/embj.2018100476)
Supplement: Supplementary file 2 — Expanded View Figures PDF [file EMBJ-38-e100476-s002.pdf]

Expanded View Figures

A

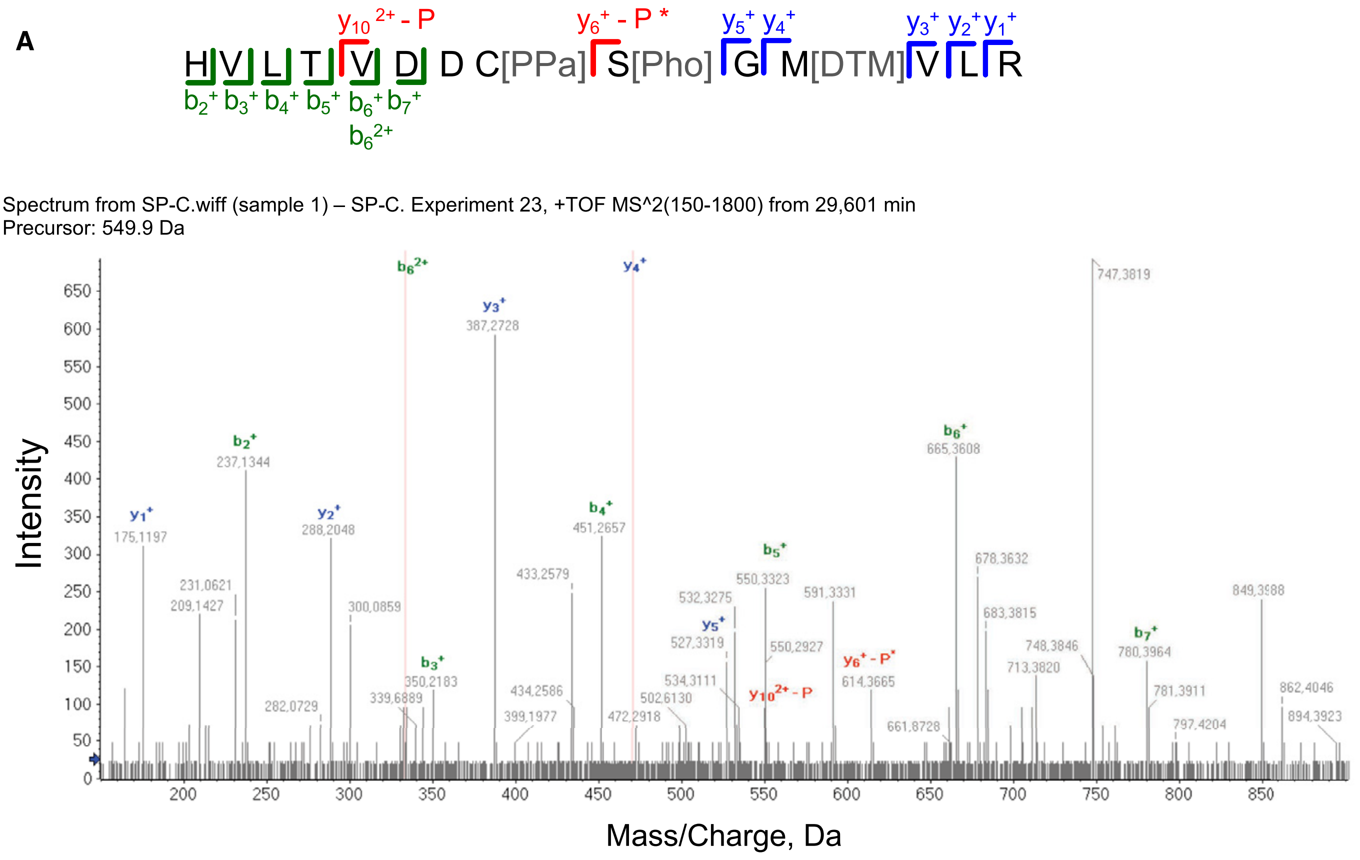

B

```
Stn1 S.pombe        1--MLENENQLTHQFPTLSRWNPMFISDVHKISFHPHLQRYIGFWMGFPIRWIQIVGYIAAI
Stn1 S.japonicus    1-MNTSAVNALVKACPTLTKWIPIFISDLYTLVRKPGV-EEVLFWYRLPVIWLRIVGIVMSV
Stn1 S.octosporus   1--MLVEDEGINKHCTTLTRWNPMFINDVYSIVFNGSTTQNIGFWFGSPVCWVQIVGYVVSL
Stn1 S.cryophilus   1--MPLEDEDINKQCTTLTRWNPMFINDVYSIVFSDSISKEIGFWFGHPICWIQVVGYVVSL
                             :  :  :    **::*  *:**.*:::.:          .  :  **     *:  *:::** :  ::

Stn1 S.pombe        60- DIYEGKHVLTVDDCSGMVLRVVFIIQDDFSMSKRAISMSPGNVVCVFGKINSFRSEVELI
Stn1 S.japonicus    61- DEYEGRTNVTVDDASGRTIVCCIDQTTNIPWTREDRSSWIGKAFRIDGRLQCIGLNRRFL
Stn1 S.octosporus   60- DVYEDRCICVVDDCTGQSLRTVFSMQEKPSLAQKASTLNPGNIVRVGGKIQRSH-SVHLV
Stn1 S.cryophilus   60- DFYEDKYVCTVDDCTSQNIRTVFSLKERRSLAQKAKRLNPGSIVRVGGKIQRVH-SFHLL
                        * ** :    .***.:.   :     :             ::.          *.  .  :  *:::      .  .::
```

Figure EV1. Identification of S74 as Stn1 phosphorylation site in fission yeast.

A Mass spectrometry spectra identifying the phosphorylation on Stn1-S74 residue.

B S74 residue is conserved within the *Schizosaccharomyces* genus. Sequence alignment of Stn1 protein in *Schizosaccharomyces* genus (*S. pombe*, *S. cryophilus*, *S. octosporus*, and *S. japonicus*) using Clustal Omega. Serine-74 is colored in red. The site is either conserved or exchanged by an amino acid capable of being phosphorylated.

 © 2019 The Authors

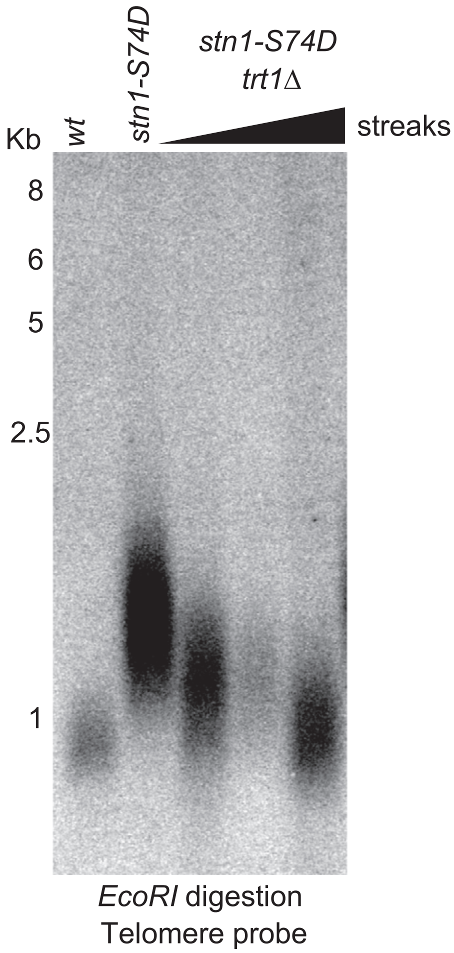

Figure EV2. Telomere length of *stn1-S74D* is dependent on telomerase.

trt1^{+} was deleted in the *stn1-S74D* background, and double mutants were streaked for multiple passages (triangle indicates increased number of generations).

© 2019 The Authors

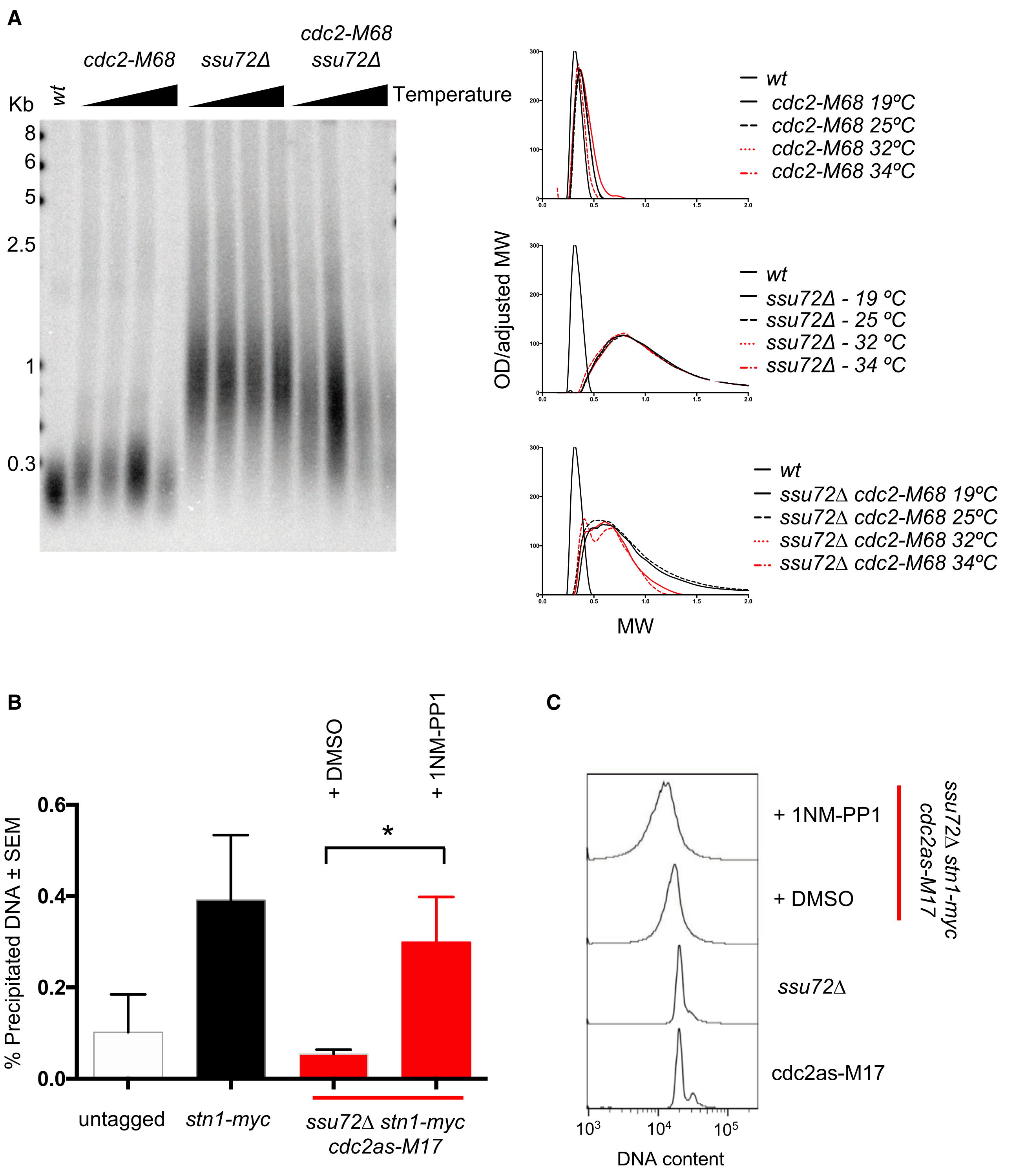

Figure EV3. Cdk1 activity counteracts Ssu72 phosphatase in telomere length regulation and Stn1 recruitment.

A *cdc2-M68 ssu72Δ* mutants grown at 25°C and shifted to different temperatures (19, 25, 32, and 34°C) for 16 h to partially inactivate *cdc2-M68* temperature-sensitive allele. DNA was isolated, and telomere length was revealed by Southern blotting using *ApaI*-digested genomic DNA.

B Densitometry and TRF analysis of Southern blots shown in (A). Quantification of 4 independent colonies $^*P \leq 0.05$ based on a two-tailed Student's *t*-test to control sample. Error bars represent standard error of the mean (SEM).

C ChIP analysis of Stn1 was carried out in *cdc2as-M17 ssu72Δ* background either treated with DMSO or using the ATP analog 1NM-PP1 (10 μM) for 3 h. Untagged and *stn1-myc* strains were used as controls. Cell cycle profiles were measured using PI FACS analysis.

 © 2019 The Authors

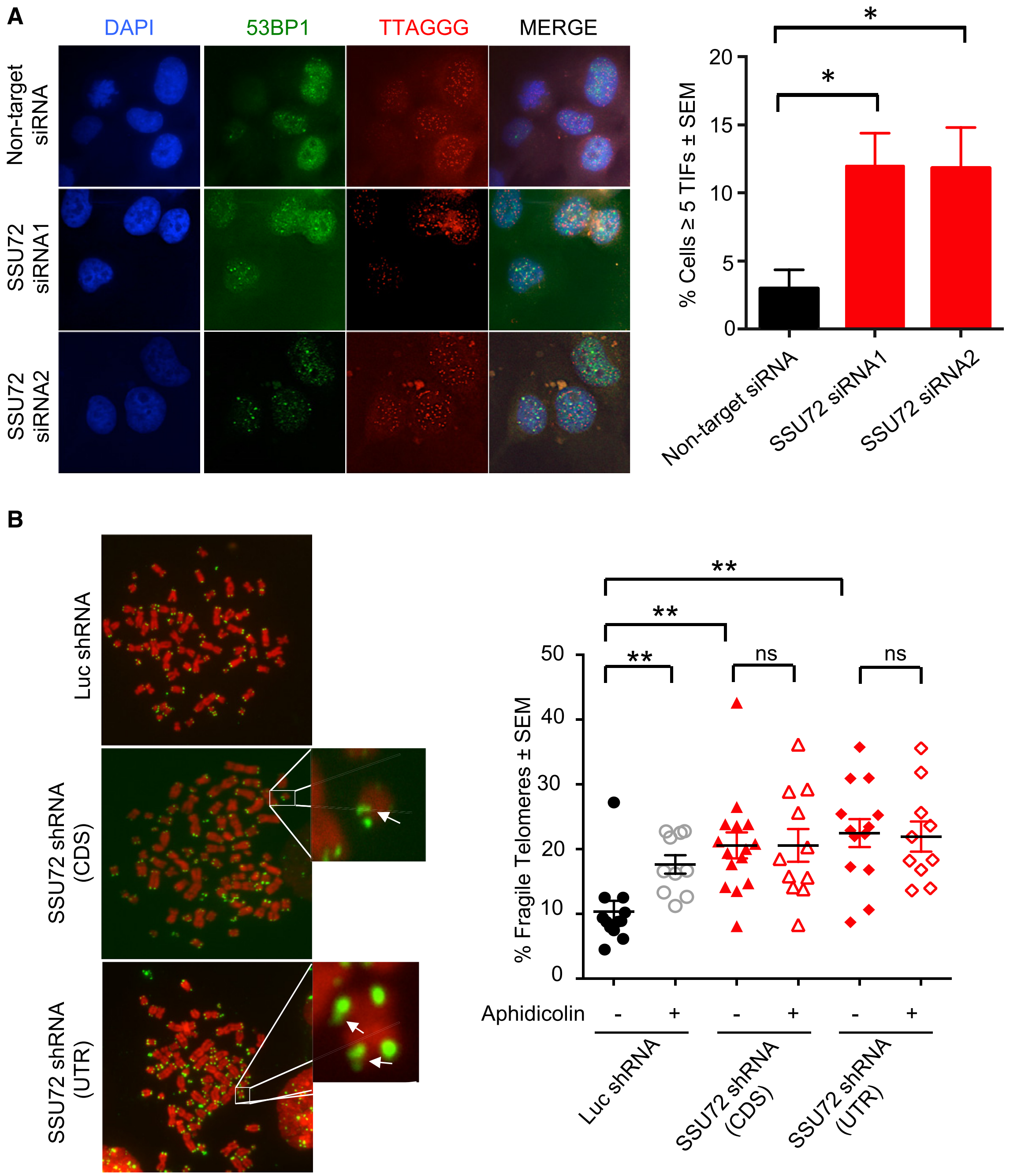

Figure EV4. SSU72 inactivation induces telomere dysfunction in different human cell lines.

A SSU72 downregulation gives rise to 53BP1 foci at telomeres in human HeLa cell line. Cells were transfected with two independent siRNAs against human SSU72 using a non-targeting siRNA as a control. After 3 days, cells were fixed and IF-FISH was performed using a 53BP1 antibody and PNA-telomere probes. Quantification of telomere-induced foci (TIF) in SSU72 downregulated cells was quantified for $n = 3$ experiments; $*P \leq 0.05$ based on a two-tailed Student's t-test to control sample. Error bars represent standard error of the mean.

B SSU72 downregulation induces telomere fragility in telomerase-negative U2OS cell lines. Cells were infected with lentiviral particles carrying shRNAs against either SSU72 or luciferase shRNAs. Cells were then treated with aphidicolin at 200 ng/ml for 12 h before colcemid treatment. Metaphases were collected, and FISH was carried out using PNA-telomere probes. MTS are represented by arrows. Quantification of MTS (per metaphase with > 6 metaphases per experimental setting for each of two independent experiments) in SSU72 downregulated cells was carried out; $**P \leq 0.01$ based on a two-tailed Student's t-test to control sample. Error bars represent standard error of the mean.

© 2019 The Authors
